# Molecular characterization and antifungal activity against non-dermatophyte molds causing onychomycosis

**DOI:** 10.1038/s41598-021-00104-0

**Published:** 2021-10-20

**Authors:** Keyvan Pakshir, Mandana Kamali, Hasti Nouraei, Kamiar Zomorodian, Marjan Motamedi, Mozhgan Mahmoodi

**Affiliations:** 1 Department of Parasitology and Mycology, School of Medicine, Shiraz University of Medical Sciences, Shiraz, Iran; 2 Basic Sciences in Infectious Diseases Research Center, School of Medicine, Shiraz University of Medical Sciences, Shiraz, Iran

**Keywords:** Microbiology, Diseases, Medical research

## Abstract

Onychomycosis is a fungal disease that caused by different types of fungi. Non-dermatophyte molds are a large saprophytic fungi group that live in nature and could affect traumatic nails. The aim of this study was to identify non-dermatophyte molds causing onychomycosis and evaluation of several antifungal activities against the isolates. The samples consisted of 50 non-dermatophyte molds isolated from patients with onychomycosis confirmed by direct and culture examination fungal. DNA was extracted, amplified, and sequenced. Disk diffusion method was used to evaluate itraconazole, fluconazole, ketoconazole, terbinafine, posaconazole, and econazole activity against the isolates. The species identified as: *Aspergillus flavus* 22 (44%), *A. niger* 12 (24%), *A. fumigates*, 3 (6%), *A. sydowii* 3 (6%), *A. terreus* 1 (2%), *Penicillium commune* 2 (4%), *P. glabrum 2* (4%), *P. chrysogenum,* 1 (2%), *Fusarium solani* 3 (6%) and *F. thapsinum* 1 (2%). Most of the samples were sensitive to terbinafine, itraconazole, and econazole and 94% of the isolates were resistant to fluconazole. This study showed that *Aspergillus* species were the most common cause of non-dermatophyte mold onychomycosis and fluconazole was the most resistant antifungals. Care must be taken to choose the appropriate antifungal drug for a better cure.

## Introduction

Fungal infections of nails is called onychomycosis and is one of the most common global nail disorder that may present on toe and fingernails, mostly on toenails^1^. This disorder included up to 18–50% of all nail diseases and 30% of cutaneous fungal infections^2^. Clinical signs included changing the color and thickening of the nail, hyperkeratosis, and onycholysis^3^. Although onychomycosis is not life-threatening, this is an important public health concern because of its high prevalence and poor response to therapy^4^. Risk factors for onychomycosis include advancing age, nail trauma or psoriasis, running or swimming, immunosuppression, diabetes, poor peripheral circulation, and fungal infection history elsewhere on the body^5,6^. Among these, trauma caused by outdoor activities in males and hand wet work in females are the major predisposing risk factors for the development of onychomycosis^7^. The tropics area with a wet humid climate that helps fungal growth, sporting, and low awareness of the mycotic disease by the general population may be a possible explanation for the high prevalence rate of onychomycosis^7^.

Onychomycosis is caused by dermatophytes, non dermatophyte (saprophytic) molds (NDMs), or yeasts^2^. The prevalence of NDMs isolated from onychomycosis in various parts of the world ranges between 1.49 and 33.5%^8,9^. This difference may be due to a difference in the geographical distribution of NDMs, diagnostic criteria, and methods for diagnosis.

The most common NDM agents are *Aspergillus* spp., *Scopulariopsis* spp., *Alternaria* spp., *Acremonium* spp., and *Fusarium* spp. that are responsible for approximately 2–25% of all the agents of onychomycosis^8,10^. *Aspergillus* spp. are increasingly being reported as primary causative agents of NDMs onychomycosis worldwide, with prevalence as high as 34.4% in Guatemala^11^, 69.3% in Iran^12^. NDMs agents especially *Aspergillus* spp. isolated from infected nails are not susceptible to most of the topical and systemic antifungals^13^.

Cure NDMs onychomycosis is difficult, and treatment often is based on oral and topical antifungal therapy while in some cases, topical antifungals may be more effective than oral antifungals, and this may depend on the causative organism^14,15^. Currently, there are five accepted classes of antifungal drugs that are related to onychomycosis: allylamines such as terbinafine, azoles such as itraconazole, morpholines, hydroxypyridinones (ciclopirox), and echinocandins^16^. The diagnosis of onychomycosis is conventionally direct microscopy and fungal culture; however, culture-based detection methods may take a few weeks for definite identification. Molecular method techniques using polymerase chain reaction (PCR) assay provide a rapid, stable, and accurate alternative for identifying pathogenic fungi in the affected nail samples of patients with onychomycosis^17^.

Onychomycosis's causative agents may vary depending upon geographic or temporal distribution, and inadequate treatment may lead to infection resistance and recurrence. Proper clinical diagnosis, laboratory workup, and adequate antifungal therapy are thus the standard of care for nails infections, so this study was aimed to find out the pattern of NDMs as causative agents of onychomycosis based on DNA sequence analysis of internal transcribed spacer (ITS) ribosomal DNA (rDNA) and evaluation of antifungal drug activities by disk diffusion methods.

## Results

**Figure 1:**
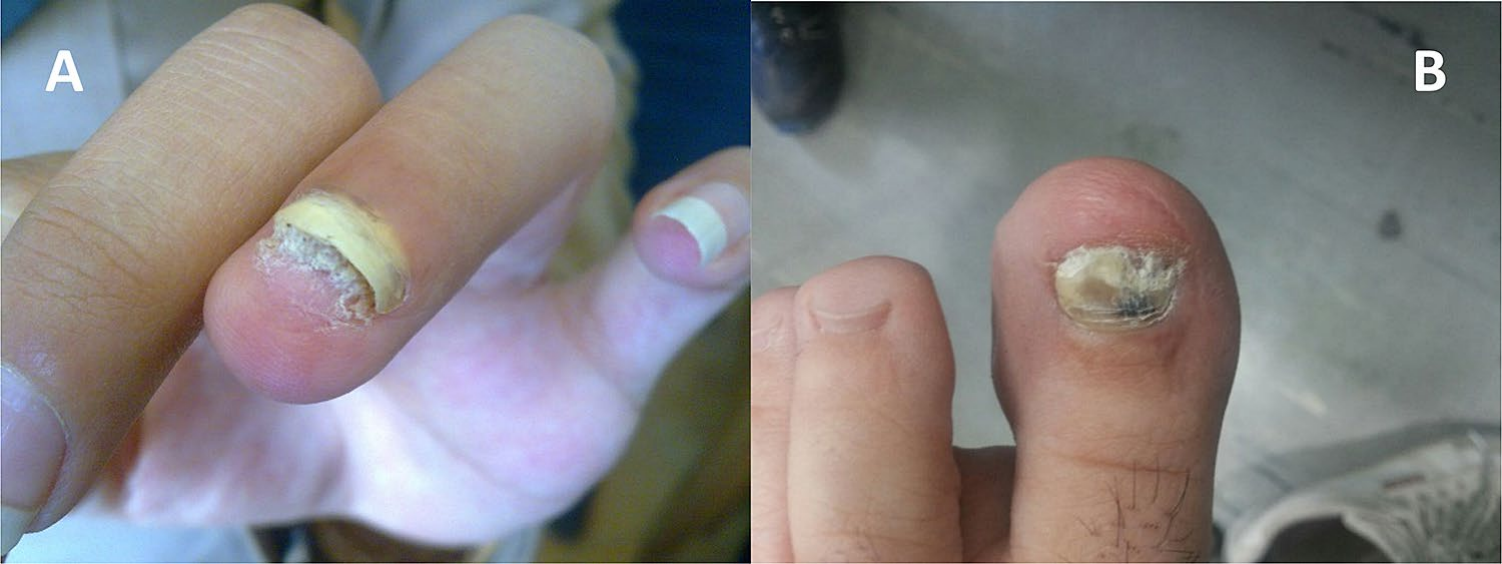
(**A**) Distal subungual onychomycosis (DSO): whitish discoloration, onycholysis and subungual hyperkeratosis. (**B**) Total dystrophy and discoloration in toenail.

In this study, the patients included 39 (78%) females and 11 (22%) males, and the samples included 39 (78%) toenails and 11 (22%) fingernails (Fig. 1).

The isolated agents and their abundance based on conventional methods were classified into three genus; *Aspergillus* spp. (82%), *Penicillium* spp. (10%) and *Fusarium* spp. (8%) (Table 1). Of 50 NDMs isolates, 16 (32%) cases did not identify at the species level by these methods.

**Table 1:** Known non-dermatophytic molds in cases of onychomycosis in this study based on culture and Sequencing methods.

Genus (number (%))	Culture method		Sequencing method	
	Species	Number (%)	Species	Number (%)
*Aspergillus* (41 (82%))	*Flavus*	22 (44%)	*Flavus*	22 (44%)
	*Niger*	12 (24%)	*Niger*	12 (24%)
	Unknown	7 (14%)	*Fumigatus*	3 (6%)
			*Sydowii*	3 (6%)
			*Terreus*	1 (2%)
*Penicillium* (5 (10%))	Unknown	5 (10%)	*Commune*	2 (4%)
			*Glabrum*	2 (4%)
			*Chrysogenum*	1 (2%)
*Fusarium* (4 (8%))	Unknown	4 (8%)	*Solani*	3 (6%)
			*Thapsinum*	1 (2%)

The BLAST analysis of the 50 DNA sequence results indicated that *Aspergillus flavus* was the most frequently species (44%), followed by *A*. *niger* (24%), *A*. *fumigatus* (6%), *A*. *sydowii* (6%), *A*. *terreus* (2%), *Penicillium commune* (4%), *P*. *glabrum* (4%), *P*. *chrysogenum* (2%), *Fusarium solani* (6%) and *F*. *thapsinum* (2%) (Table 1).

**Figure 2:**
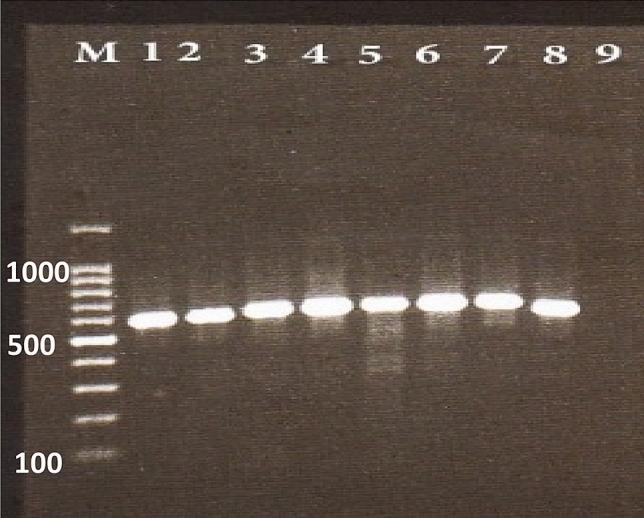
Representative agarose gel electrophoresis of PCR products. Lanes 1, 2: *Penicillium* spp; Lane 3: *Aspergillus* sp; Lanes 4: *Fusarium* sp; Lanes 5–8: *Aspergillus* spp; lane 9: negative controls and lanes M is the 100-bp molecular size marker.

The results of culture and sequencing indicate that the three genus of *Aspergillus, Penicillium,* and *Fusarium* were identified equally in both methods. Two species of *flavus* and *niger* were identified equally at the species level, but the other unknown species could identify by the sequencing method (Fig. 2).

**Figure 3:**
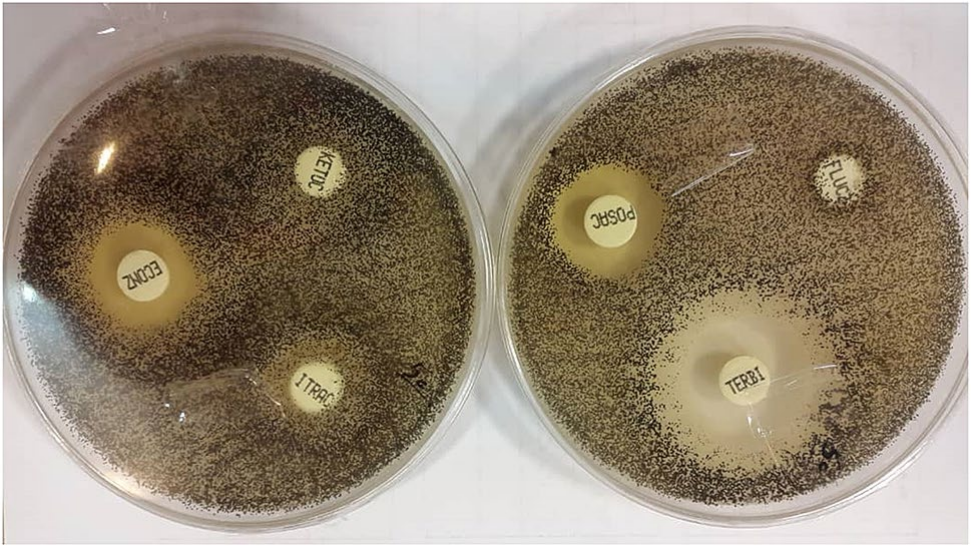
Antifungal sensitivity of *Asperigillus niger* showing high sensitivity to terbinafine and resistance to fluconazole, ketoconazole and itraconazole.

The antifungal drugs patterns (susceptible, intermediate, and resistant) against NDMs is shown in Table 2. The highest sensitivity was observed in terbinafine, itraconazole, and econazole, respectively. Of all these sensitive isolates, two cases were intermediate to terbinafine and econazole, and 1 case was resistant, and 1 case was intermediate to itraconazole. Forty-two isolates (84%) were sensitive to posaconazole, and the remaining were resistant (16%). The results showed that 36 isolates (72%) were sensitive to ketoconazole, 13 isolates (26%) were intermediate, and one isolate (2%) was resistant (Fig. 3).

**Table 2:** Antifungal susceptibility pattern of non-dermatophytic molds disk diffusion method.

Antifungal drugs	Number	Pattern	Genus		
			*Aspergillus*	*Penicillium*	*Fusarium*
Posaconazole	42(84%)	S	36(87.8%)	4(80%)	2(50%)
	**-**	I	**-**	**-**	-
	8(16%)	R	5(12.2%)	1(20%)	2(50%)
Itraconazole	48(96%)	S	40(97.56%)	5(100%)	3(75%)
	1(2%)	I	**-**	-	1(25%)
	1(2%)	R	1(2.44%)	-	-
Fluconazole	1(2%)	S	-	-	1(25%)
	2(4%)	I	-	2(40%)	-
	47(94%)	R	41(100%)	3(60%)	3(75%)
Terbinafine	48(96%)	S	41(100%)	5(100%)	2(50%)
	2(4%)	I	-	-	2(50%)
	-	R	-	-	-
Ketoconazole	36(72%)	S	30(73.8%)	5(100%)	2(50%)
	13(26%)	I	11(26.82%)	-	2(50%)
	1(2%)	R	-	-	-
Econazole	48(96%)	S	39(95.13%)	5(100%)	3(75%)
	2(4%)	I	2(4.87%)	-	1(25%)
	-	R	-	-	-

S: susceptible, I: intermediate, R: resistant.

Based on the criteria of sensitivity and resistance, the highest resistance was observed in fluconazole, and it was statistically significant among other drugs (*p*-value < 0.001).

It is noteworthy that 47 out of 50 cases (94%) were resistant to fluconazole, and only 1 case was sensitive to this drug, and the remaining two were intermediate.

In three genus, 1 *Aspergillus*, 2 *Penicillium,* and 2 *Fusarium* isolates were resistant to posaconazole and fluconazole. There was only one isolate resistant to itraconazole, which was related to *A. flavus* species (Table 2).

Also, the resistance samples to posaconazole compared to itraconazole and ketoconazole were significant. The *p*-value in both was 0.036, so the resistance of the samples to posaconazole was higher than itraconazole and ketoconazole.

The most effective drugs were terbinafine, itraconazole, and econazole that 96% of the samples were sensitive. The next most effective drugs were ketoconazole and posaconazole, which 84% and 72% of the samples were sensitive to them, respectively. Sensitive samples to econazole, itraconazole, and terbinafine compared to ketoconazole were statistically significant with a *p*-value of 0.001, so samples' sensitivity to econazole, itraconazole, and terbinafine were higher than ketoconazole. There was no significant difference in the samples' sensitivity to ketoconazole, econazole, itraconazole, and terbinafine compared to posaconazole (*p*-value > 0.05). The susceptibility of most frequent species (*A. flavus* and *A. niger*) to all drugs except ketoconazole and fluconazole was the same (*p*-value > 0.05), but ketoconazole was found to be more effective in *flavus* species than niger species (*p*-value < 0.001). Both species were determined to have a fluconazole-resistant ratio.

## Discussion

Onychomycosis is one of the most common nail infections in which dermatophytes, yeasts, and non dermatophyte molds have been incriminated as etiological agents. According to the latest reports, the global prevalence of onychomycosis is nearly 2–9%^7^. Onychomycosis often was mimicked with psoriasis of the nail, eczema, bacterial infections, and contact dermatitis^18^. So, correctly determining the etiologic agents of onychomycosis is important to provide a baseline for administering appropriate antifungal therapy and identifying the source of infection, hence facilitating prevention measures^19^.

At the moment, traditional mycology remains the gold standard for diagnosing NDMs onychomycosis. This includes obtaining positive results from KOH and culture. The culture method, although it demonstrates the viability and allows the identification of isolated fungi but it is time-consuming, and due to the diversities and similarities among different species, morphological methods are not specific enough. It appears that a more accurate method is critical to differentiate various members of the genus^20^.

In addition to standard procedures, the use of molecular biological techniques such as polymerase chain reaction (PCR), followed by sequencing of appropriate targets, has important implications for understanding these pathogenic fungi^21^. *Scopulariopsis brevicaulis*, *Fusarium* species, *Aspergillus* species, *Neoscytalidium dimidiatum*, and *Acremonium* species are the most common causative agents of onychomycosis in the world that we could not isolate some of them such as *S. brevicaulis*, *N. dimidiatum* or *Acremonium* spp.^13^. Comparison of culture and molecular methods indicated that *Aspergillus*, *Penicillium,* and *Fusarium* genus were identified in both methods. Using molecular methods increased the sensitivity and specificity of identification of microorganisms that caused onychomycosis as our 16 unknown isolates were identified by this method as followed: *A. fumigatus* (n = 3), *A. sydowii* (n = 3), *A. terreus* (n = 1), *Penicillium commune* (n = 2), *P. glabrum* (n = 2), *P. chrysogenum* (n = 1), *Fusarium solani* (n = 3) and *F. thapsinum* (n = 1).

In this study, *A. flavus* was responsible for approximately 44% of all the samples and that this data was agree with Nouripour-Sisakht et al.^22^, Chadeganipour et al.^23^ and Halvaee et al.^24^, they reported that 77.3%, 66% and 60.9% of their isolates were *A. flavus,* respectively. The high frequency of nail infections due to *A. flavus* can be attributed to the fact that dry climate in Iran that favors the growth of thermo-tolerant fungi like *A. flavus* and this species is one of the most frequent *Aspergillus* species isolated from the environment^25,26^. This genus is one of the common pathogenic fungi in humans^27^. Also, in Hashemi et al. study, based on conventional method, *A. flavus* reported as predominant agent responsible for NDMs onychomycosis infections^28^. Our study was in contrast with Martinez et al. in Brazil^11^ and Bitew et al. in Ethiopia^7^ that reported *A. niger* and *Neoscytalidium dimidiatum*, respectively, as more prevalent species. Differences in patient characteristics, sample size, and study design could be reasons for such discrepancy.

*Penicillium commune* and *P. glabrum* that identified were reported for the first time from onychomycosis patients in Iran but also those fungi have been isolated from environmental area of Mazandaran province in north of Iran previously and, identified by beta-tubulin gene sequencing method^29^. It is hard to identify these organism by conventional methods maybe for this reason there was less reports regarding these isolates from onychomycosis around the world. *Penicillium chrysogenum* as pathogenic fungi is involved in developing skin diseases such as onychomycosis and invasive diseases^30^.

Treatment is necessary for onychomycosis to avoid serious consequences such as secondary bacterial infections, complete nail dystrophy, and cosmetic reasons^31^. It is including use topical and oral agents and physical treatments^32^. Drug choice depends on several things such as the severity of nail damage, type of organism, adverse effects, and success or failure of previous treatments^33^.

Treatment in NDMs onychomycosis is dependent on fungal species that caused infection and their susceptibility to antifungals prescription because different fungi have different responses to drugs. On the other hand, many of the selected therapies were failed because of increasing the prevalence of drug resistance related to the fungi genetic factors and the type of antifungals or host conditions^33^.

Drug susceptibility tests have several benefits, such as preventing long-term, unnecessary, and even high-risk drug use and reducing treatment costs^34^.

The disk diffusion method is a simple, inexpensive, and rapid method that qualitatively demonstrates the antifungal drugs' effectiveness^35^. In our study, terbinafine, econazole, and itraconazole were identified as the most effective antifungal drugs against onychomycosis caused by NDMs agents. In fact, out of the 50 isolates, 96% of them were sensitive to these three antifungals.

Except for several cases of *Fusarium solani* and *Aspergillus flavus* all the isolates were sensitive to terbinafine, econazole and itraconazole.

As shown in our in vitro study, terbinafine and itraconazole were the best candidates for NDM onychomycosis treatment, but it needs more details about in vivo data analysis. One reason for the good effects of terbinafine is its appropriate penetration into the nail matrix^36^.

As we showed in this study, all *Aspergillus* species were sensitive to terbinafine, and this result agreed with Gupta et al.^13^ and Trovato et al.^37^ in Italy and Haghani et al.^38^ and Xu et al.^25^ in Iran. In other study in Iran reported that some *Aspergillus* species were resistance to terbinafine and this result was not agree with our study^39^.

A noteworthy point in this study was the high resistance of isolates to fluconazole (94%). All species of *Aspergillus* were resistant to fluconazole. Our results agreed with Tsang et al.^40^ study and were in contrast with Haghani et al.^38^ that reported *Aspergillus terreus* was sensitive to fluconazole. So, fluconazole is not suitable to cure onychomycosis causing by NDMs but is commonly prescribed in combination with the other antifungals such as itraconazole and terbinafine cases of moderate to severe nail involvement. The results in Falahati et al. study showed that the clinical isolate of *A. clavatus* resistant to the wide spectrum of antifungal drugs such as terbinafine, posaconazole, voriconazole, etc. but sensitive to itraconazole^39^. Due to the side effects and high resistance of NDMs to fluconazole, drug susceptibility testing is important^31^.

It is necessary to mention that two isolates of *Fusarium solani* were resistant to all five antifungal drugs, except each one of them was sensitive to econazole and itraconazole separately. Bueno et al. also reported *Fusarium* as resistant fungi to antifungal drugs in his study^41^. Although *Fusarium* had less frequency than the other genus in our study, special attention should be given to these genus because of their antifungal resistance and keratinolytic activity that helped *Fusarium* to use nails as a source of nutrition^42^.

The high prevalence rate of onychomycosis with different fungi groups involving etiological agents suggests that more studies should be done on the prevalence. This is for better understanding these issues and could cause developing better preventive procedures and reducing treatment costs. Also, in vivo studies are needed to confirm the effects of drugs in order to find a suitable drug to treat these cases of onychomycosis.

## Conclusion

Onychomycosis is a common multifactorial disease of the nails. The prevalence of fungal nail infections is affected by age, lifestyle, and some underlying diseases. A global outlook on the treatment of onychomycosis provides a framework of success for the clinician. Healthcare could be more effective if physicians prescribe the most effective antifungals for the treatment of onychomycosis.

## Materials and methods

### Collection of nail samples

Fifty NDMs onychomycosis cases among 300 clinically suspected patients with onychomycosis referred to medical mycology laboratory enrolled in this study. Patients who had a previous history of antifungal therapy were excluded from the study. Small pieces of the nail debris were collected from the affected area using sterile nail clippers and scalpel.

### Mycological examination

A part of nail scrapings was immersed in a drop of 20% KOH over a slide and kept in a wet chamber at room temperature for direct microscopy examination.

The other parts of the nail samples were simultaneously inoculated on Sabouraud's dextrose agar (SDA) (Merck, Darmstadt, Germany), SDA containing chloramphenicol (0.05 mg/l), with and without actidione (500 mg/l). The cultures were incubated at 28 °C for 1–2 weeks. The plates were examined twice weekly for any growth. No growth at the second week was considered as negative. A broad septate/non septate hyphae detection in the direct exam along with positive culture (saprophyte colony growth) was considered as NMD onychomycosis. Genus and species of fungi were identified by colony morphology and microscopic examination by using teas mounts and slide culture. Diagnosis of NDM onychomycosis was made on the basis of the following criteria: (1) nail abnormalities; (2) positive KOH, (3) isolation in culture, (4) repeated isolation in culture (5) failure to isolate a dermatophyte in culture; and in suspicious cases we checked growth of more than 5 colonies of the same mold in at least 2 repeated nail samplings^43,44^.

### Preparation of genomic DNA

Total cellular DNA was extracted from a small amount of mycelium cultured on Sabouraud agar slants, by the rapid mini-preparation method^45^. Briefly, to a 1.5 ml eppendorf tube containing 500 µl of lysis buffer (400 mM Tris–HCl [pH = 8], 60 mM EDTA pH [pH = 8], 150 mM NaCl, 1% [w:v] SDS), a small portion of mycelium grown on SDA in primary culture was added using a sterile toothpick, with which the mycelia were disrupted. The tube was then left at room temperature for 10 min. After adding 150 µl of potassium acetate buffer pH [pH = 4.8] (5 M potassium acetate, acetic acid), the tube was vortexed briefly, and cellular debris and precipitated proteins were removed by centrifugation at > 10,000 g for 1 min. The supernatant was transferred to another 1.5 ml eppendorf tube and centrifuged again as above. After the supernatant was transferred to a new 1.5 ml eppendorf tube, an equal volume of isopropyl alcohol was added. The tube was mixed briefly by inversion, centrifuged at > 10,000 g for 2 min, and the supernatant was discarded. The resultant DNA pellet was washed in 300 ml of 70% (v:v) ethanol. After centrifuging at 10,000 g for 1 min, the supernatant was discarded. The DNA pellet is air-dried and dissolved in 50 µl of DW.

### Polymerase chain reaction (PCR) amplification

A 6 µl aliquot of template DNA, 0.5 µM of each forward (ITS1: 5′-TTC GTA GGT GAA CCT GCG G-3′) and reverse primer (ITS4: 5′-TCC TCC GCT TAT TGA TAT GC-3′), 25 µl of premix (Ampliqon, Denmark) and final volume of 50 µl were used for PCR, under the following conditions: initial denaturation for 5 min at 94 °C; 32 cycles for denaturation (30 s at 94 °C); annealing for 45 s at 56 °C; and extension for 45 s at 72 °C, followed by an ultimate extension step at 72 °C for 7 min. The PCR products were electrophoresed on a 1.2% agarose gel.

### Sequencing and sequence analysis

All the amplified PCR products were sequenced unilaterally, using the forward primer (ITS1) as for the primary PCR, via an automated DNA sequencer (Bioneer Company, South Korea). The sequences were edited with Geneious software (www.geneious.com), and for final identification, the obtained consensus sequences were compared with the pubmed database (https://www.ncbi.nlm.nih.gov/pubmed/).

### Antifungal susceptibility

Antifungal susceptibility test by disk diffusion method was done according to CLSI M 51-A document. In brief, the isolates were subcultured on potato dextrose agar (PDA) at 25 °C for 2–7 days before testing. The conidial inocula were prepared, and the turbidity was adjusted by spectrophotometry to an optical density of 0.09 to 0.13. Then entire surface of mueller hinton agar (MHA) was inoculated with a sterile cotton swab with the undiluted mold stock inoculum suspension. Antifungal disks (Rosco Diagnostica, Denmark) including ketoconazol (15 µg), fluconazole (25 µg), itraconazole (10 µg), posaconazole (5 µg), terbinafine (1 µg) and econazole (1 µg) were applied to the inoculated media. The plates were incubated at 35 °C and read after 2–3 days. Inhibition zone diameter were interpreted in accordance with CLSI M51-A (Table 3).

**Table 3:** Inhibition zones diameters (mm) of antifungal tablets examined in this study after inoculation of non-dermatophytic mold suspension.

Antifungal tablets	Potency (µg)	Zone diameter in mm		
		Sensitive	Intermediate	Resistant
Ketoconazol	15	≥ 28	27 – 21	≤ 20
Fluconazole	25	≥ 19	15 – 18 (DD)	≤ 14
Itraconazole	10	≥ 23	14 – 22 (DD)	≤ 13
Posaconazole	5	≥ 17	14 – 16 (DD)	≤ 13
Econazole	1	≥ 20	12 – 19	≤ 11
Terbinafine	1	≥ 20	12 – 19	≤ 11

DD = doses dependent.

### Statistical analysis

Results were analyzed using the SPSS22 (Statistical Package for the Social Sciences) program. Fisher exact test and chi-square were considered statistically significant with a *p*-value less than 0.05.

### Ethical statement

This project was found to be according to the ethical principles and the national norms and standards for conducting Medical Research in Iran and has been approved by the research ethics committee (Shiraz University of Medical Sciences. IR.SUMS.REC.1396.5775).

### Informed consent

Informed consent to participate and publish was obtained from all the participants.

## Data Availability

The data used to support the findings of this study were supplied by the Vice-Chancellor for Research of Shiraz University of Medical Sciences under license. Requests for data access should be made to Keyvan Pakshir, pakshirk@gmail.com.
